# Paradoxical Psoriasis Induced by Anti-TNFα Treatment: Evaluation of Disease-Specific Clinical and Genetic Markers

**DOI:** 10.3390/ijms21217873

**Published:** 2020-10-23

**Authors:** Agostino Bucalo, Federica Rega, Arianna Zangrilli, Valentina Silvestri, Virginia Valentini, Giorgia Scafetta, Federica Marraffa, Sara Grassi, Elena Rogante, Arianna Piccolo, Salvatore Cucchiara, Franca Viola, Luca Bianchi, Laura Ottini, Antonio Richetta

**Affiliations:** 1 Department of Molecular Medicine, Sapienza University of Rome, 00161 Rome, Italy; agostino.bucalo@uniroma1.it (A.B.); valentina.silvestri@uniroma1.it (V.S.); virginia.valentini@uniroma1.it (V.V.); giorgia.scafetta@uniroma1.it (G.S.); 2 Clinic of Dermatology Department of Clinical Internal, Anesthesiological and Cardiovascular Sciences, Sapienza University of Rome, 00161 Rome, Italy; rega.federica@alice.it (F.R.); federica.marraffa@uniroma1.it (F.M.); sara.grassi03@gmail.com (S.G.); elenarogante@virgilio.it (E.R.); antonio.richetta@uniroma1.it (A.R.); 3 Dermatology Unit, Policlinico Tor Vergata, Tor Vergata University of Rome, 00133 Rome, Italy; arianna.zangrilli@libero.it (A.Z.); ari.piccolo91@gmail.com (A.P.); luca.bianchi@uniroma2.it (L.B.); 4 Pediatric Gastroenterology and Liver Unit, Maternal and Child Health Department, Sapienza University of Rome, 00161 Rome, Italy; salvatore.cucchiara@uniroma1.it (S.C.); franca.viola@uniroma1.it (F.V.)

**Keywords:** paradoxical psoriasis, inflammatory bowel disease, psoriasis, tumor necrosis factor-alpha inhibitors, genetic polymorphisms

## Abstract

Paradoxical psoriasis (PP) may occur during treatment with anti-tumor necrosis factor-alpha (TNF-α) drugs in various chronic immune-mediated diseases, mainly inflammatory bowel diseases (IBD) and psoriasis. In this study, clinical and genetic characteristics of PP arising in IBD and psoriatic patients were investigated to identify disease-specific markers of the paradoxical effect. A total of 161 IBD and psoriatic patients treated with anti-TNF-α drugs were included in the study. Of these patients, 39 developed PP. All patients were characterized for the main clinical–pathologic characteristics and genotyped for six candidate single nucleotide polymorphisms (SNPs) selected for their possible role in PP susceptibility. In IBD patients, the onset of PP was associated with female sex, presence of comorbidities, and use of adalimumab. IBD patients with PP had a higher frequency of the *TNF-α* rs1799964 rare allele (*p* = 0.006) compared with cases without the paradoxical effect, and a lower frequency of the human leucocyte antigen (*HLA*)-*Cw06* rs10484554 rare allele (*p* = 0.03) compared with psoriatic patients with PP. Overall, these findings point to specific clinical and genetic characteristics of IBD patients with PP and provide data showing that genetic variability may be related to the paradoxical effect of anti-TNF-α drugs with possible implications into clinical practice.

## 1. Introduction

Paradoxical psoriasis (PP) represents a peculiar type of psoriasis that may occur de novo or as the exacerbation of pre-existent psoriatic lesions during treatment with biological drugs [1]. Specifically, its onset is observed during treatment with tumor necrosis factor-alpha (TNF-α) inhibitors, such as infliximab, adalimumab, and etanercept [1,2,3,4].

The first case of PP induced by anti -TNF-α treatment was described in a patient affected by inflammatory bowel diseases (IBD) more than 15 years ago [5], and later, an increasing number of cases of PP has been reported in the literature, mainly due to the widespread use of anti-TNF-α drugs [6,7,8,9,10,11].

For their good safety profile and high sustainability, TNF-α inhibitors have revolutionized the management of numerous chronic immune-mediated inflammatory diseases, including IBD and psoriasis [12,13,14,15]. However, like other immunomodulatory drugs, TNF-α inhibitors are associated with adverse reactions, among which is the occurrence of PP [1,3,4,11,12,16,17,18].

PP is observed in about 2–5% of patients treated with anti-TNF-α drugs, with a slight predilection for women [3,19,20,21,22]. The time between the onset of PP and the introduction of the treatment can range from a few days to several months [3]. The most frequently reported clinical presentations of PP are palmoplantar pustular eruption, plaque-type and guttate psoriasis, and nail and scalp involvement have also been described [23,24,25,26].

Notably, numerous diseases that benefit from TNF-α inhibitor therapy are associated with an inherent increased risk of classical psoriasis. In particular, IBD and psoriasis are associated conditions, and the prevalence of psoriasis among patients affected by IBD is higher (6–10%) compared to the general population (2%) [27].

Although PP clinically may resemble classical psoriasis, there is evidence that it represents a distinct pathological entity [28], and different underlying pathogenic mechanisms may be involved in classical and paradoxical psoriasis. Classical psoriasis is a T-cell mediated autoimmune disease driven by TNF, while in contrast, PP is caused by the absence of TNF and represents a type-I interferon (IFN)-driven innate inflammation [29]. Indeed, TNF controls the production of type I-IFN by plasmacitoid dendritic cells (pDCs), and TNF blockade induces its continuous overexpression driving paradoxical effect [30].

Predictor factors of PP onset are largely unknown. Recently, Ya et al. showed that the presence of family history (FH) of classical psoriasis is associated with the onset of PP, thus suggesting a possible role of genetic predisposition in the development of PP [31].

Common polymorphisms, particularly those within Interleukin 23 Receptor (*IL23R*) gene, have been proposed to be involved in PP [23,26,32]. Interestingly, the *IL23R* rs11209026 polymorphism was reported as a risk factor for PP in IBD patients [32] in contrast to a protective role reported in classical psoriasis [33]. On the other hand, the human leucocyte antigen (*HLA*)-*Cw0602* allele, the allele most frequently associated with classical psoriasis [34], has been rarely reported in PP [16]. Overall, these findings may suggest genetic differences between classical and paradoxical psoriasis.

Considering the critical role of TNF and type-I IFN in the pathogenesis of classical and paradoxical psoriasis, polymorphisms in these genes might play a role in the pathogenesis of the two diseases. Polymorphisms in the *Interferon Induced with Helicase C Domain 1* (IFIH1) gene, a gene encoding a cytoplasmic viral RNA receptor that activates type-I IFN signaling, are considered risk factors for various autoimmune diseases, including classical psoriasis [23,30,35,36]. Moreover, polymorphisms in the *TNF-α* promoter, such as rs1799964 and rs1800629, are known to be involved in modulating anti-TNF-α response in classical psoriasis and IBD [14,37,38].

Aims of this study were to investigate and compare clinical and genetic characteristics of PP arising in IBD and psoriatic patients during anti-TNF-α treatment in order to identify disease-specific markers of the paradoxical effect. To this purpose, IBD and psoriatic patients, treated with anti-TNF-α drugs and characterized for the main clinical–pathologic characteristics, were genotyped at six single nucleotide polymorphisms (SNPs) selected for their possible role in the susceptibility to classical and paradoxical psoriasis and in the response to anti-TNF-α drugs.

## 2. Results

### 2.1. Clinical-Pathologic Characteristics of the Patients Analyzed

The clinical–pathologic characteristics of IBD and psoriatic patients are detailed in Table 1 and Table 2, respectively. As shown in Table 1, the majority of IBD patients were males (56.5%), had a diagnosis of Crohn’s disease (69.8%), did not show FH for IBD (79.3%), did not present comorbidities (90.6%), and were treated with infliximab (77.4%). IBD patients with PP significantly differed from IBD patients without PP in relation to sex (*p* < 0.001), presence of comorbidities (*p* = 0.01), and the biological drug used (*p* < 0.001). The majority of IBD cases with PP were females and showed comorbidities, including asthma, allergy, and osteoporosis. Specifically, four of the five IBD patients with comorbidities who developed PP were females. Although the infliximab was the most widely used anti-TNF-α drug, the PP was most frequently observed in patients treated with adalimumab.

As shown in Table 2, the majority of psoriatic patients were males (66.7%), had a severe type of psoriasis (83.2%), (65.3%), particularly hypercholesterolemia, hypertension, and diabetes, and were treated with adalimumab (86.1%). Psoriatic patients with and without PP did not significantly differ in relation to the clinical–pathologic features considered. Notably, all psoriatic patients who developed PP were treated with adalimumab.

Clinical–pathologic characteristics, including sex, number of lesion locations, lesion location, presence of pruritus, and time elapsed between the start of the treatment with adalimumab and the onset of PP were compared in IBD and psoriatic patients (Table S1). A statistically significant difference between IBD and psoriatic emerged for the number of lesion locations (*p* = 0.003), lesion location (*p* = 0.04), and timing of PP onset (*p* = 0.009). Specifically, compared with psoriatic patients, IBD patients showed a more severe paradoxical effect in terms of greater number of locations (63.6% vs. 15.8%) and more frequently showed scalp lesions (25.8% vs. 3.7%). Pruritus was more frequent in psoriatic patients compared with IBD patients (*p* = 0.05). Furthermore, the mean time elapsed between the start of therapy and the paradoxical effect development was significantly lower (*p* = 0.009) in IBD patients than in psoriatic patients (9.0 vs. 40.8 months).

### 2.2. Genotyping Analysis

All 161 patients were genotyped for six SNPs, including *HLA-Cw0602* rs10484554, *IL23R* rs11209026, and rs10789229, *TNF-α* rs1799964, and rs1800629 and *IFIH1* rs1990760.

Firstly, we compared the distribution of genotype frequencies of the six SNPs in IBD patients with and without PP. As shown in Table 3, a statistically significant difference in the distribution of genotypes emerged for *TNF-α* rs1799964 (*p* = 0.008). IBD cases with PP had a higher probability to be carriers of the *TNF-α* rs1799964 rare C allele (OR 5.3; 95% CI 1.6–17.2; *p* = 0.006) compared with IBD patients without the paradoxical effect. No statistically significant differences emerged for any of the polymorphisms analyzed when we compared psoriatic patients with and without PP (Table S2).

When we compared the distribution of genotype frequencies of the six SNPs in IBD patients with PP and psoriatic patients without PP (Table 4), statistically significant differences in the distribution of genotypes emerged for *HLA-Cw06* rs10484554 (*p* = 0.02), *IL23R* rs10789229 (*p* = 0.01), and *TNF-α* rs1799964 (*p* = 0.04). IBD cases with PP had a lower probability to be carriers of the *HLA-Cw06* rs10484554 rare T allele (OR 0.2; 95% CI 0.1–0.7 *p* = 0.01) and a higher probability to be carriers of the *TNF-α* rs1799964 rare C allele (OR 3.0; 95% CI 1.2–7.5; *p* = 0.02), compared with psoriatic cases without paradoxical effect. No statistically significant results emerged for *IL23R* rs10789229 (OR 0.7; 95% CI 0.9–1.5) *p* = 0.3). Similar results were obtained when analyses were performed including only patients in treatment with adalimumab (Table S3).

We also compared the distribution of the genotype frequencies of the six SNPs in IBD and psoriatic patients with PP including only cases treated with adalimumab (Table 5). A statistically significant difference in the distribution of genotypes emerged for *HLA-Cw06* rs10484554 (*p* = 0.05). IBD cases with PP had a lower probability to be carriers of the *HLA-Cw06* rs10484554 rare T allele (OR 0.2; 95% CI 0.04–0.8; *p* = 0.03) compared with psoriatic patients with PP.

## 3. Discussion

PP is a particular type of psoriasis induced by anti-TNF-α drugs that may occur either de novo, or as an aggravation of pre-existent lesions [1]. PP is generally reported during anti-TNF-α treatment, such as infliximab and adalimumab, in diseases for which anti-TNF-α treatment is indicated, particularly IBD and psoriasis [1,9,16,17,23,32,39,40,41,42,43,44]. In this study, analyzing a large series of IBD and psoriatic patients with and without PP, we were able to identify disease-specific, clinical, and genetic characteristics of the paradoxical effect, and to highlight the differences between paradoxical and classical psoriasis.

Our results showed that the majority of IBD patients with PP were females, had comorbidities, and were treated with adalimumab. Female gender has been associated with higher risk of developing PP, and it may be considered a risk factor for PP. Hormonal or biological drug dosing-based influences may explain this association [3,19,20,21,22,25]. In our series, the majority of female IBD patients with PP and comorbidities were affected mainly by comorbidities such as asthma and allergies, thus suggesting a major dysregulation of the immune system in female PP patients.

Adalimumab treatment was associated with PP onset in IBD patients, although infliximab was the anti-TNF-α drug most frequently used in this setting. These findings are consistent with recent studies showing a stronger association of PP with adalimumab treatment [45,46,47,48,49,50,51], in contrast to the initially reported association with infliximab therapy [17,42,45,46]. In our study, psoriatic patients who developed PP were also in treatment with adalimumab. However, since adalimumab was the anti-TNF-α drug most frequently used in these patients, we cannot draw any conclusion on possible differences in drug effects on developing PP in this setting. Our data also show that the time interval between the beginning of adalimumab treatment and the onset of the paradoxical effect was shorter in IBD patients compared with psoriatic patients. The interval time of about nine months observed for IBD patients in our study is comparable to the median latency of 11 months, reported in a previous study on anti-TNF-α induced PP [25].

Overall, our findings suggest disease-specific features of PP. Compared with psoriatic patients, IBD patients with PP showed a more severe paradoxical effect, in terms of greater number of locations, and frequent scalp lesions. The finding that significant scalp involvement occurred more often in IBD patients is consistent with previous published data [25,40,44] and supports the idea that IBD patients may have distinctive genetic/immunologic factors predisposing to scalp PP development.

We evaluated possible associations between genetic factors and PP predisposition by genotyping IBD and psoriatic patients for 6 SNPs, including *HLA-Cw0602* rs10484554, *IL23R* rs11209026, and rs10789229, *TNF-α* rs1799964, and rs1800629 and *IFIH1* rs1990760, selected for their possible role in PP [23,32,34,35,36,37,52]. Overall, IBD patients with PP had a higher frequency of the *TNF-α* rs1799964 rare C allele and a lower frequency of the *HLA-Cw06* rs10484554 rare T allele.

The *TNF-α* rs179996 rare C allele was more frequent in IBD patients with PP, compared to both IBD and psoriatic patients without paradoxical effect, and was associated with a higher risk of PP. In contrast, no differences in genotype frequencies of the *TNF-α* rs179996 rare C allele emerged comparing psoriatic patients with and without PP. These results suggest that the *TNF-α* rs179996 rare C allele may represent a genetic risk factor predisposing to PP specifically in IBD patients.

The *TNF-α* rs179996 corresponds to a T/C transition at nucleotide position -1031, in the promoter region of the *TNF-α* gene. It has been shown that psoriatic patients with the wild-type T allele are more likely to respond to anti-TNF-α drugs compared with carriers of the rare C allele [46]. Furthermore, there is evidence that the C/C genotype may cause an increased expression of *TNF-α* and may represent a possible genetic risk factor for IBD [38]. In the present study, we identified the *TNF-α* rs179996 rare C as a possible genetic risk factor for PP in IBD patients. We can hypothesize that in IBD patients, the *TNF-α* rs179996 rare C allele, particularly in the homozygote genotype, may cause an increase of *TNF-α* expression and a reduction of response to anti-TNF-α therapy, thus predisposing to the paradoxical adverse reaction in patients treated with anti-TNF-α drugs, especially adalimumab [38,46]. Further studies are needed to explore this hypothesis.

The *HLA-Cw06* rs10484554 rare T allele was significantly less frequent in IBD patients compared with psoriatic patients. The *HLA-Cw06* rs10484554 rare T allele is the allele most frequently associated with classical psoriasis [34,52]. A previous study showed that this allele was rare in IBD patients with anti-TNF-α-induced psoriatic lesions [53]. Our results provide evidence that *HLA-Cw06* rs10484554 rare T is not associated with PP and support the hypothesis that the PP may be related to a different genetic background compared to classical psoriasis [16,30]. The absence of differences between psoriatic patients with and without PP may indicate that other SNPs, yet to be identified, may play a role in predisposing to the paradoxical effect in psoriatic patients. However, as we observed a considerably long time (40 months) between the start of anti-TNF therapy and PP development in psoriatic patients, we cannot exclude that some patients may have experienced a relapse of the disease (i.e., a secondary failure of anti-TNF) rather than a PP.

In conclusion, this study adds strength to the current evidence showing that psoriasis and PP are two different entities, at pathological, clinical, and genetic levels. Moreover, the paradoxical effect may have disease-specific features, such as the number and location of psoriatic lesions. The *TNF-α* rs179996 rare C allele may be a predisposing genetic factor to PP in IBD patients, particularly those in treatment with adalimumab. The *HLA-Cw06* rs10484554 is a main predisposing genetic factor for classical psoriasis, but not for PP, further supporting their different pathogenesis. Overall, our findings point to specific clinical and genetic characteristics of IBD patients with PP and provide data showing that genetic variability may be related to the paradoxical effect of anti-TNF-α drugs with possible implications into clinical practice. Further studies are needed to investigate the possible role of other candidate SNPs in the predisposition to this intriguing adverse reaction.

## 4. Materials and Methods

### 4.1. Sample Collection and DNA Extraction

**Figure 1:**
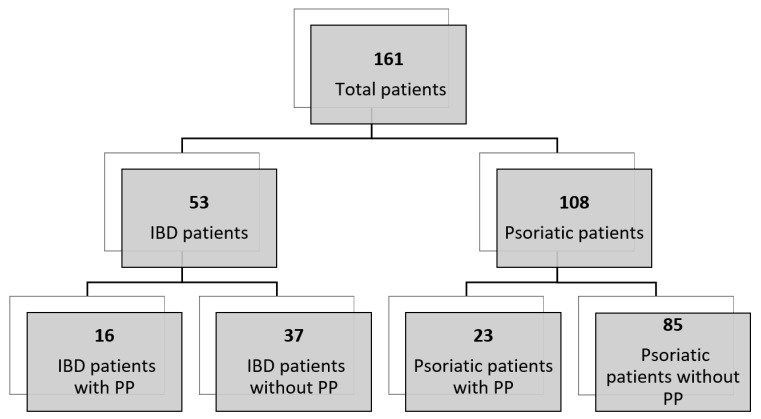
Diagram showing the number of patients analyzed in the present study.

A total of 161 patients were recruited for the present study (Figure 1). The inclusion criteria for all patients were a) being in treatment with an anti-TNFα drug; b) being diagnosed with IBD or classical psoriasis. Overall, 53 IBD patients and 108 psoriatic patients were included in the study.

The cohort of 53 IBD patients, recruited at the Complex Operative Unit of Gastroenterology, Epatology and Pediatric Digestive Endoscopy of Policlinico Umberto I, Rome, included a retrospective cohort of 16 IBD patients with a clinical diagnosis of PP and a consecutive cohort of 37 IBD patients without PP, enrolled between November 2017 and November 2018. IBD patients with a diagnosis of psoriasis before starting the anti-TNFa therapy were excluded.

The cohort of 108 psoriatic patients, recruited at the Complex Operative Unit of Dermatology, Department of Internal Medicine and Medical Specialties, of Policlinico Umberto I and at the Department of Dermatology, University Hospital of Tor Vergata, Rome, included a retrospective cohort of 23 psoriatic patients with a clinical diagnosis of PP [47], and a consecutive cohort of 85 patients with classical psoriasis, enrolled between November 2017 and December 2019. Psoriatic patients with a concomitant diagnosis of both classical psoriasis and IBD were excluded.

A signed informed consent form, with a detailed description of the study protocol, was collected for each study participant. The study was approved by The Local Ethical Committee (Sapienza University of Rome 26/09/2013, Protocol 873/13).

For all cases, the main personal and clinical–pathologic data, including sex, age at diagnosis of disease, family history, comorbidities, therapy, and pathology were collected. In addition, information about the severity of psoriasis and location of psoriatic lesions, presence of pruritus, and time elapsed (in months) between the start of biological treatment and the development of PP were collected. Severity of psoriasis was assessed based on Psoriasis Area Severity Index (PASI) and Dermatology Life Quality Index (DLQI) scores. Psoriasis severity was defined using the following criteria: Mild, PASI < 7 and DLQI < 7; moderate, PASI = 7–15 and DLQI = 5–15; severe, PASI > 15, independently of the DLQI score [54].

Blood samples were obtained for each study participant. DNA from blood samples was extracted using ReliaPrep Blood gDNA Miniprep System (Promega, Madison, WI, USA), according to the manufacturer’s instructions. DNA quantification was performed with Qubit 2.0 dsDNA BR Assay Kit (Invitrogen, Carlsbad, CA, USA), according to the manufacturer’s instructions.

### 4.2. Genotyping Analysis

All enrolled cases were genotyped for six polymorphisms, selected for their possible role in the susceptibility to classical and paradoxical psoriasis and in the response to anti-TNF-α drugs, including: *HLA-Cw0602* rs10484554, *IL23R* rs11209026, and rs10789229, *TNF-α* rs1799964, and rs1800629 and *IFIH1* rs1990760 [14,23,32,33,34,35,36,37,38,46,52,55,56,57]. Genotyping was performed by allelic discrimination real-time PCR, on the ABI7500 fast real-time PCR instrument (Life Technologies, Carlsbad, CA, USA), using commercially available TaqMan SNP genotyping assays (IDT-TEMA Coralville, IA, USA) and according to the manufacturer’s instructions. In each experiment, positive and negative controls were also included.

### 4.3. Statistical Analysis

Possible associations between selected clinical–pathologic features and specific groups of cases were assessed by chi-square and T-test where appropriate.

The genotype frequencies for each polymorphism were evaluated in each group of patients and differences were evaluated by chi-square test. The association between polymorphisms and disease status was estimated using univariate logistic regression and was measured by the odds ratio (OR) and its corresponding 95% confidence interval (CI). For each polymorphism, we evaluated ORs based on multiplicative codominant (per-allele) model. A *p*-value <0.05 was considered statistically significant. All analyses were performed using STATA version 13.1 statistical program (StataCorp).

## Figures and Tables

**Table 1:** Clinical–pathologic characteristics of inflammatory bowel diseases (IBD) patients with and without paradoxical psoriasis (PP).

Characteristics ^a^	Total (*n* = 53)	IBD Patients with PP (*n* = 16)	IBD Patients without PP (*n* = 37)	*p* Value *
**Sex**				
Male	30 (56.5%)	3 (18.8%)	27 (73.0%)	
Female	23 (43.4%)	13 (81.3%)	10 (27.0%)	**<0.001**
**Mean age at diagnosis** (Mean ± Standard error)	11.0 (±0.5)	10.5 (±0.7)	11.2 (±0.6)	0.5
**Type of IBD**				
Crohn’s disease	37 (69.8%)	14 (87.5%)	23 (62.2%)	
Ulcerative colitis	16 (30.2%)	2 (12.5%)	14 (37.8%)	0.07
**Family history for IBD**				
Yes	11 (20.7%)	4 (25.0%)	7 (18.9%)	
No	42 (79.3%)	12 (75.0%)	30 (81.1%)	0.6
**Comorbidities**				
Yes	5 (9.4%)	4 (25.0%)	1 (2.7%)	
No	48 (90.6%)	12 (75.0%)	36 (97.3%)	**0.01**
**Biological drug**				
adalimumab	12 (22.6%)	11 (68.7%)	1 (2.7%)	
infliximab	41 (77.4%)	5 (31.3%)	36 (97.3%)	**<0.001**

* In bold are statistically significant results. ^a^ Some data for each pathologic characteristic are not available.

**Table 2:** Clinical–pathologic characteristics of psoriatic patients with and without (PP).

Characteristics ^a^	Total (*n* = 108)	Psoriatic Patients with PP (*n* = 23)	Psoriatic Patients without PP (*n* = 85)	*p* Value *
**Sex**				
Male	72 (66.7%)	14 (60.9%)	58 (68.2%)	
Female	36 (33.3%)	9 (39.1%)	27 (31.8%)	0.5
**Mean age at diagnosis** (Mean ± Standard error)	30.6 (±1.5)	25.6 (±2.9)	32 (±1.7)	0.08
**Degree of psoriasis**				
Mild	8 (7.5%)	0 (0.0%)	8 (9.5%)	
Moderate	10 (9.3%)	0 (0.0%)	10 (11.9%)	
Severe	89 (83.2%)	23 (100%)	66 (78.6%)	*0.05*
**Arthropathic psoriasis**				
Yes	60 (55.6%)	14 (60.9%)	46 (54.1%)	
No	48 (44.4%)	9 (39.1%)	39 (45.9%)	0.6
**Family history for psoriasis**				
Yes	50 (51.0%)	11 (47.8%)	39 (52.0%)	
No	48 (49.0%)	12 (52.2%)	36 (48.0%)	0.7
**Comorbidities**				
Yes	64 (65.3%)	16 (69.6%)	48 (64.0%)	
No	34 (34.7%)	7 (30.4%)	27 (36.0%)	0.6
**Biological drug**				
adalimumab	93 (86.1%)	23 (100%)	70 (82.4%)	
infliximab	3 (2.8%)	0 (0.0%)	3 (3.5%)	
etanercept	8 (7.4%)	0 (0.0%)	8 (9.4%)	
golimumab	4 (3.7%)	0 (0.0%)	4 (4.7%)	0.2

^a^ Some data for each pathologic characteristic are not available. * In bold are statistically significant results.

**Table 3:** Distribution of IBD patients with and without PP according to the genotype frequencies of the six single nucleotide polymorphisms (SNPs) analyzed.

Gene	SNP	Genotype	IBD Patients with PP (*n* = 16)	IBD Patients without PP (*n* = 37)	*p* Value *
			n (%)	n (%)	
***HLA-Cw06***	**rs10484554**	C/C	12 (75.0%)	28 (75.7%)	0.6
		C/	4 (25.0%)	7 (18.9%)	
		T/T	0 (0.0%)	2 (5.4%)	
		Per-allele	OR 0.8 (95% CI 0.3–2.6) *p* = 0.8		
***IL23R***	**rs11209026**	G/G	12 (75.0%)	34 (91.9%)	0.09
		G/A	4 (25.0%)	3 (8.1%)	
		A/A	0 (0.0%)	0 (0.0%)	
		Per-allele	OR 3.8 (95% CI 0.7–19.4) *p* = 0.1		
***IL23R***	**rs10789229**	T/T	11 (68.7%)	21 (56.8%)	0.2
		T/C	1 (6.3%)	10 (27.0%)	
		C/C	4 (25.0%)	6 (16.2%)	
		Per-allele	OR 0.9 (95% CI 0.4–2.0) *p* = 0.9		
***TNF±***	**rs1799964**	T/T	5 (31.2%)	26 (70.3%)	**0.008**
		T/C	9 (56.3%)	11 (29.7%)	
		C/C	2 (12.5%)	0 (0.0%)	
		Per-allele	OR 5.3 (95% CI 1.6–17.2) ***p* = 0.006**		
***TNF±***	**rs1800629**	G/G	16 (100%)	35 (94.6 %)	0.3
		G/A	0 (0.0%)	2 (5.4%)	
		A/A	0 (0.0%)	0 (0.0%)	
		Per allele	-		
***IFIH1***	**rs1990760**	T/T	7 (43.7)	15 (40.5%)	0.6
		T/C	7 (43.8%)	13 (35.1%)	
		C/C	2 (12.5%)	9 (24.3%)	
		Per allele	OR 0.8 (95% CI 0.3–1.7) *p* = 0.5		

* In bold are statistically significant results.

**Table 4:** Distribution of IBD patients with PP and psoriatic patients without PP according to the genotype frequencies of the six SNPs analyzed.

Gene	SNP	Genotype	IBD Patients with PP (*n* = 16)	Psoriatic Patients without PP (*n* = 85)	*p* Value *
			n (%)	n (%)	
***HLA-Cw06***	**rs10484554**	C/C	12 (75.0%)	34 (40.0%)	**0.02**
		C/T	4 (25.0%)	41 (48.2%)	
		T/T	0 (0.0%)	10 (11.8%)	
		Per-allele	OR 0.2 (95%CI 0.1–0.7) ***p* = 0.01**		
***IL23R***	**rs11209026**	G/G	12 (75.0%)	74 (87.1%)	0.2
		G/A	4 (25.0%)	11 (12.9%)	
		A/A	0 (0.0%)	0 (0.0%)	
		Per-allele	OR 2.2 (95%CI 0.6–8.2) *p* = 0.2		
***IL23R***	**rs10789229**	T/T	11 (68.7%)	33 (38.8%)	**0.01**
		T/C	1 (6.3%)	40 (47.1%)	
		C/C	4 (25.0%)	12 (14.1%)	
		Per-allele	OR 0.7 (95%CI 0.9–1.5) *p* = 0.3		
***TNF±***	**rs1799964**	T/T	5 (31.2%)	50 (58.8%)	**0.04**
		T/C	9 (56.3%)	33 (38.8%)	
		C/C	2 (12.5%)	2 (2.4%)	
		Per-allele	OR 3 (95%CI 1.2–7.5) ***p* = 0.02**		
***TNF±***	**rs1800629**	G/G	16 (100%)	77 (90.6%)	0.4
		G/A	0 (0.0%)	7 (8.2%)	
		A/A	0 (0.0%)	1 (1.2%)	
		Per-allele	-		
***IFIH1***	**rs1990760**	T/T	7 (43.7%)	29 (34.1%)	0.5
		T/C	7 (43.8%)	34 (40.0%)	
		C/C	2 (12.5%)	22 (25.9%)	
		Per-allele	OR 0.7 (95%CI 0.3–1.4) *p* = 0.3		

* In bold are statistically significant results.

**Table 5:** Distribution of IBD and psoriatic patients with PP treated with adalimumab according to the genotype frequencies of the six SNPs analyzed.

Gene	SNP	Genotype	IBD Patients with PP (*n* = 11)	Psoriatic Patients with PP (*n* = 23)	*p* Value *
			n (%)	n (%)	
***HLA-Cw06***	**rs10484554**	C/C	9 (81.8%)	9 (39.1%)	**0.05**
		C/T	2 (18.2%)	9 (39.1%)	
		T/T	0 (0.0%)	5 (21.8%)	
		Per-allele	OR 0.2 (95%CI 0.04–0.8) ***p* = 0.03**		
***IL23R***	**rs11209026**	G/G	9 (81.8%)	22 (95.6%)	0.18
		G/A	2 (18.2%)	1 (4.4%)	
		A/A	0 (0.0%)	0 (0.0%)	
		Per-allele	OR 4.9 (95%CI 0.4–60.9) *p* = 0.2		
***IL23R***	**rs10789229**	T/T	8 (72.7%)	11 (47.8%)	0.08
		T/C	0 (0.0%)	8 (34.8%)	
		C/C	3 (27.3%)	4 (17.4%)	
		Per-allele	OR 0.8 (95%CI 0.3–2) *p* = 0.6		
***TNF±***	**rs1799964**	T/T	4 (36.4%)	13 (56.5%)	0.55
		T/C	5 (45.5%)	7 (30.4%)	
		C/C	2 (18.1%)	3 (13.1%)	
		Per-allele	OR 1.6 (95% CI 0.6–4.3) *p* = 0.3		
***TNF±***	**rs1800629**	G/G	11 (100%)	21 (91.3%)	0.6
		G/A	0 (0.0%)	1 (4.4%)	
		A/A	0 (0.0%)	1 (4.3%)	
		Per-allele	-		
***IFIH1***	**rs1990760**	T/T	4 (36.3%)	8 (34.8%)	1
		T/C	5 (45.5%)	11 (47.8%)	
		C/C	2 (18.2%)	4 (17.4%)	
		Per-allele	OR 1 (95%CI 0.3–2.7) *p* = 1		

* In bold are statistically significant results.

## Supplementary Materials

Supplementary materials can be found at https://www.mdpi.com/1422-0067/21/21/7873/s1.

## Abbreviations

PP	Paradoxical psoriasis
TNF-α	Tumor necrosis factor-alpha
IBD	Inflammatory bowel diseases
IFN	Interferon
pDCs	Plasmacytoid dendritic cells
FH	Family history
IL23R	Interleukin 23 Receptor
*HLA*	Human leucocyte antigen
IFIH1	Interferon induced with helicase C domain 1
SNPs	Single nucleotide polymorphisms
PASI	Psoriasis area severity index
DLQI	Life quality index
OR	Odds ratio
CI	Confidence interval

## References

[1] Puig L. (2018). Paradoxical Reactions: Anti-Tumor Necrosis Factor Alpha Agents, Ustekinumab, Secukinumab, Ixekizumab, and Others. Curr. Probl. Derm..

[2] Ciccarelli F., De Martinis M., Sirufo M.M., Ginaldi L. (2016). Psoriasis Induced by Anti-Tumor Necrosis Factor Alpha Agents: A Comprehensive Review of the Literature. Acta Derm. Croat..

[3] Brown G., Wang E., Leon A., Huynh M., Wehner M., Matro R., Linos E., Liao W., Haemel A. (2016). Tumor Necrosis Factor-α Inhibitor-Induced Psoriasis: Systematic Review of Clinical Features, Histopathological Findings, and Management Experience. J. Am. Acad. Derm..

[4] Collamer A.N., Guerrero K.T., Henning J.S., Battafarano D.F. (2008). Psoriatic Skin Lesions Induced by Tumor Necrosis Factor Antagonist Therapy: A Literature Review and Potential Mechanisms of Action. Arthritis Rheum..

[5] Dereure O., Guillot B., Jorgensen C., Cohen J.D., Combes B., Guilhou J.J. (2004). Psoriatic lesions induced by anti-tumour necrosis factor-alpha treatment: Two cases. Br. J. Derm..

[6] George L.A., Gadani A., Cross R.K., Jambaulikar G., Ghazi L.J. (2015). Psoriasiform Skin Lesions Are Caused by Anti-TNF Agents Used for the Treatment of Inflammatory Bowel Disease. Dig. Dis. Sci..

[7] Denadai R., Teixeira F.V., Steinwurz F., Romiti R., Saad-Hossne R. (2013). Induction or Exacerbation of Psoriatic Lesions during Anti-TNF-α Therapy for Inflammatory Bowel Disease: A Systematic Literature Review Based on 222 Cases. J. Crohns Colitis.

[8] Cullen G., Kroshinsky D., Cheifetz A.S., Korzenik J.R. (2011). Psoriasis associated with anti-tumour necrosis factor therapy in inflammatory bowel disease: A new series and a review of 120 cases from the literature. Aliment. Pharm..

[9] Pugliese D., Guidi L., Ferraro P.M., Marzo M., Felice C., Celleno L., Landi R., Andrisani G., Pizzolante F., De Vitis I. (2015). Paradoxical Psoriasis in a Large Cohort of Patients with Inflammatory Bowel Disease Receiving Treatment with Anti-TNF Alpha: 5-Year Follow-up Study. Aliment. Pharm..

[10] Rahier J.F., Buche S., Peyrin-Biroulet L., Bouhnik Y., Duclos B., Louis E., Papay P., Allez M., Cosnes J., Cortot A. (2010). Severe skin lesions cause patients with inflammatory bowel disease to discontinue anti-tumor necrosis factor therapy. Clin. Gastroenterol. Hepatol..

[11] Guerra I., Pérez-Jeldres T., Iborra M., Algaba A., Monfort D., Calvet X., Chaparro M., Mañosa M., Hinojosa E., Minguez M. (2016). Incidence, Clinical Characteristics, and Management of Psoriasis Induced by Anti-TNF Therapy in Patients with Inflammatory Bowel Disease: A Nationwide Cohort Study. Inflamm. Bowel Dis..

[12] Andrisani G., Marzo M., Celleno L., Guidi L., Papa A., Gasbarrini A., Armuzzi A. (2013). Development of Psoriasis Scalp with Alopecia during Treatment of Crohn’s Disease with Infliximab and Rapid Response to Both Diseases to Ustekinumab. Eur. Rev. Med. Pharm. Sci..

[13] Lis K., Kuzawińska O., Bałkowiec-Iskra E. (2013). Tumor necrosis factor inhibitors—State of knowledge. Arch. Med. Sci..

[14] Gallo E., Cabaleiro T., Román M., Solano-López G., Abad-Santos F., García-Díez A., Daudén E. (2013). The Relationship between Tumour Necrosis Factor (TNF)-α Promoter and *IL12B*/*IL-23R* Genes Polymorphisms and the Efficacy of Anti-TNF-α Therapy in Psoriasis: A Case-Control Study. Br. J. Derm..

[15] Pagnini C., Pizzarro T.T., Cominelli F. (2019). Novel Pharmacological Therapy in Inflammatory Bowel Diseases: Beyond Anti-Tumor Necrosis Factor. Front. Pharm..

[16] Melo F.J., Magina S. (2018). Clinical Management of Anti-TNF-Alpha-Induced Psoriasis or Psoriasiform Lesions in Inflammatory Bowel Disease Patients: A Systematic Review. Int. J. Derm..

[17] Cleynen I., Vermeire S. (2012). Paradoxical Inflammation Induced by Anti-TNF Agents in Patients with IBD. Nat. Rev. Gastroenterol. Hepatol..

[18] Bae J.M., Lee H.H., Lee B.I., Lee K.M., Eun S.H., Cho M.L., Kim J.S., Park J.M., Cho Y.S., Lee I.S. (2018). Incidence of Psoriasiform Diseases Secondary to Tumour Necrosis Factor Antagonists in Patients with Inflammatory Bowel Disease: A Nationwide Population-Based Cohort Study. Aliment. Pharm..

[19] Baeten D., Kruithof E., Van den Bosch F., Van den Bossche N., Herssens A., Mielants H., De Keyser F.F., Veys E.M. (2003). Systematic safety follow up in a cohort of 107 patients with spondyloarthropathy treated with infliximab: A new perspective on the role of host defence in the pathogenesis of the disease?. Ann. Rheum. Dis..

[20] Sfikakis P.P., Iliopoulos A., Elezoglou A., Kittas C., Stratigos A. (2005). Psoriasis induced by anti-tumor necrosis factor therapy: A paradoxical adverse reaction. Arthritis Rheum..

[21] Cohen J.D., Bournerias I., Buffard V., Paufler A., Chevalier X., Bagot M., Claudepierre P. (2007). Psoriasis induced by tumor necrosis factor-alpha antagonist therapy: A case series. J. Rheumatol..

[22] De Gannes G.C., Ghoreishi M., Pope J., Russell A., Bell D., Adams S., Shojania K., Martinka M., Dutz J.P. (2007). Psoriasis and pustular dermatitis triggered by TNF- {alpha} inhibitors in patients with rheumatologic conditions. Arch. Derm..

[23] Cabaleiro T., Prieto-Pérez R., Navarro R., Solano G., Román M., Ochoa D., Abad-Santos F., Daudén E. (2015). Paradoxical Psoriasiform Reactions to Anti-TNFα Drugs Are Associated with Genetic Polymorphisms in Patients with Psoriasis. Pharm. J..

[24] Ko J.M., Gottlieb A.B., Kerbleski J.F. (2009). Induction and exarcerbation of psoriasis with TNF-blockade therapy: A review and analysis of 127 cases. J. Dermatol. Treat..

[25] Mazloom S.E., Yan D., Hu J.Z., Ya J., Husni M.E., Warren C.B., Fernandez A.P. (2018). TNF-α Inhibitor-Induced psoriasis: A decade of experience at the Cleveland Clinic. J. Am. Acad. Derm..

[26] Vedak P., Kroshinsky D., St John J., Xavier R.J., Yajnik V., Ananthakrishnan A.N. (2016). Genetic basis of TNF-α antagonist associated psoriasis in inflammatory bowel diseases: A genotype-phenotype analysis. Aliment. Pharm..

[27] Eppinga H., Poortinga S., Thio H.B., Nijsten T.E.C., Nuij V.J.A.A., van der Woude C.J., Vodegel R.M., Fuhler G.M., Peppelenbosch M.P. (2017). Prevalence and Phenotype of Concurrent Psoriasis and Inflammatory Bowel Disease. Inflamm. Bowel Dis..

[28] Hu J.Z., Billings D.S., Yan D., Fernandez A.P. (2020). Histologic comparison of tumor necrosis factor-α inhibitor-induced psoriasis and psoriasis vulgaris. J. Am. Acad. Derm..

[29] Mylonas A., Conrad C. (2018). Psoriasis: Classical vs. Paradoxical. The Yin-Yang of TNF and Type I Interferon. Front. Immunol..

[30] Conrad C., Di Domizio J., Mylonas A., Belkhodja C., Demaria O., Navarini A.A., Lapointe A.K., French L.E., Vernez M., Gilliet M. (2018). TNF Blockade Induces a Dysregulated Type I Interferon Response without Autoimmunity in Paradoxical Psoriasis. Nat. Commun..

[31] Ya J., Hu J.Z., Nowacki A.S., Khanna U., Mazloom S., Kabbur G., Husni M.E., Fernandez A.P. (2020). Family history of psoriasis, psychological stressors, and tobacco use are associated with the development of tumor necrosis factor-α inhibitor-induced psoriasis: A case-control study. J. Am. Acad. Derm..

[32] Sherlock M.E., Walters T., Tabbers M.M., Frost K., Zachos M., Muise A., Pope E., Griffiths A.M. (2013). Infliximab-Induced Psoriasis and Psoriasiform Skin Lesions in Pediatric Crohn Disease and a Potential Association with IL-23 Receptor Polymorphisms. J. Pediatr. Gastroenterol. Nutr..

[33] Di Meglio P., Di Cesare A., Laggner U., Chu C.C., Napolitano L., Villanova F., Tosi I., Capon F., Trembath R.C., Peris K. (2011). The IL23R R381Q Gene Variant Protects against Immune-Mediated Diseases by Impairing IL-23-Induced Th17 Effector Response in Humans. PLoS ONE.

[34] Mallon E., Bunker C.B., Newson R. (1999). HLA-Cw6 and the Genetic Predisposition to Psoriasis: A Meta-Analysis of Published Serologic Studies. J. Invest. Derm..

[35] Cen H., Wang W., Leng R.X., Wang T.Y., Pan H.F., Fan Y.G., Wang B., Ye D.Q. (2013). Association of IFIH1 Rs1990760 Polymorphism with Susceptibility to Autoimmune Diseases: A Meta-Analysis. Autoimmunity.

[36] Chen G., Zhou D., Zhang Z., Kan M., Zhang D., Hu X., Feng G., Liu Y., He L. (2012). Genetic Variants in IFIH1 Play Opposite Roles in the Pathogenesis of Psoriasis and Chronic Periodontitis: Genetic Variants in IFIH1. Int. J. Immunogenet..

[37] Song G.G., Seo Y.H., Kim J.H., Choi S.J., Ji J.D., Lee Y.H. (2015). Association between *TNF-α* (-308 A/G, -238 A/G, -857 C/T) Polymorphisms and Responsiveness to TNF-α Blockers in Spondyloarthropathy, Psoriasis and Crohn’s Disease: A Meta-Analysis. Pharmacogenomics.

[38] Nourian M., Chaleshi V., Pishkar L., Azimzadeh P., Baradaran Ghavami S., Balaii H., Alinaghi S., Shahrokh S., Asadzadeh Aghdaei H., Zali M.R. (2017). Evaluation of Tumor Necrosis Factor (TNF)-α MRNA Expression Level and the Rs1799964 Polymorphism of the TNF-α Gene in Peripheral Mononuclear Cells of Patients with Inflammatory Bowel Diseases. Biomed. Rep..

[39] Kirthi S., Tobin A.M., Hussey M., Scaldaferri F., McNamara D. (2017). Anti-tnfα antibody induced psoriasiform skin lesions in patients with inflammatory bowel disease: An irish cohort study. QJM.

[40] Eickstaedt J.B., Killpack L., Tung J., Davis D., Hand J.L., Tollefson M.M. (2017). Psoriasis and Psoriasiform Eruptions in Pediatric Patients with Inflammatory Bowel Disease Treated with Anti-Tumor Necrosis Factor Alpha Agents. Pediatr. Derm..

[41] Viola F., Civitelli F., Di Nardo G., Barbato M.B., Borrelli O., Oliva S., Conte F., Cucchiara S. (2009). Efficacy of adalimumab in moderate-to-severe pediatric Crohn’s disease. Am. J. Gastroenterol..

[42] Hiremath G., Duffy L., Leibowitz I. (2011). Infliximab-induced psoriasis in children with inflammatory bowel disease. J. Pediatr. Gastroenterol. Nutr..

[43] Costa-Romero M., Coto-Segura P., Suarez-Saavedra S., Ramos-Polo E., Santos-Juanes J. (2008). Guttate psoriasis induced by infliximab in a child with Crohn’s disease. Inflamm. Bowel Dis..

[44] Perman M.J., Lovell D.J., Denson L.A., Farrell M.K., Lucky A.W. (2012). Five cases of anti-tumor necrosis factor alpha-induced psoriasis presenting with severe scalp involvement in children. Pediatr. Derm..

[45] Nuti F., Viola F., Civitelli F., Alessandri C., Aloi M., Dilillo A., Del Giudice E., Cucchiara S. (2014). Biological Therapy in a Pediatric Crohn Disease Population at a Referral Center. J. Pediatr. Gastroenterol. Nutr..

[46] Ovejero-Benito M.C., Muñoz-Aceituno E., Reolid A., Saiz-Rodríguez M., Abad-Santos F., Daudén E. (2018). Pharmacogenetics and Pharmacogenomics in Moderate-to-Severe Psoriasis. Am. J. Clin. Derm..

[47] Zangrilli A., Bavetta M., Mazzilli S., Garofalo V., Bianchi L. (2018). Paradoxical Case Effects of Psoriasis Following Adalimumab Therapy: A Case Series. Dermatology.

[48] Weizman A.V., Sharma R., Afzal N.M., Xu W., Walsh S., Stempak J.M., Nguyen G.C., Croitoru K., Steinhart A.H., Silverberg M.S. (2018). Stricturing and Fistulizing Crohn’s Disease Is Associated with Anti-Tumor Necrosis Factor-Induced Psoriasis in Patients with Inflammatory Bowel Disease. Dig. Dis. Sci..

[49] Baumgart D.C., Grittner U., Steingräber A., Azzaro M., Philipp S. (2011). Frequency, phenotype, outcome, and therapeutic impact of skin reactions following initiation of adalimumab therapy: Experience from a consecutive cohort of inflammatory bowel disease patients. Inflamm. Bowel Dis..

[50] Tanaka H., Kamata N., Yamada A., Endo K., Fujii T., Yoshino T., Sugaya T., Yokoyama Y., Bamba S., Umeno J. (2018). Long-term retention of adalimumab treatment and associated prognostic factors for 1189 patients with Crohn’s disease. J. Gastroenterol. Hepatol..

[51] Mattozzi C., Richetta A.G., Cantisani C., Giancristoforo S., D’Epiro S., Gonzalez Serva A., Viola F., Cucchiara S., Calvieri S. (2010). Morphea, an unusual side effect of anti-TNF-alpha treatment. Eur. J. Derm..

[52] Singh S., Pradhan D., Puri P., Ramesh V., Aggarwal S., Nayek A., Jain A.K. (2019). Genomic Alterations Driving Psoriasis Pathogenesis. Gene.

[53] Mälkönen T., Wikström A., Heiskanen K., Merras-Salmio L., Mustonen H., Sipponen T., Kolho K.L. (2014). Skin reactions during anti-TNFα therapy for pediatric inflammatory bowel disease: A 2-year prospective study. Inflamm. Bowel Dis..

[54] Llamas-Velasco M., de la Cueva P., Notario J., Martínez-Pilar L., Martorell A., Moreno-Ramírez D. (2017). Moderate Psoriasis: A Proposed Definition. Actas Dermosifiliogr..

[55] Abdollahi E., Tavasolian F., Momtazi-Borojeni A.A., Samadi M., Rafatpanah H. (2016). Protective Role of R381Q (Rs11209026) Polymorphism in *IL-23R* Gene in Immune-Mediated Diseases: A Comprehensive Review. J. Immunotoxicol..

[56] Xu W.D., Xie Q.B., Zhao Y., Liu Y. (2015). Association of Interleukin-23 receptor gene polymorphisms with susceptibility to Crohn’s disease: A meta-analysis. Sci. Rep..

[57] Peng L.L., Wang Y., Zhu F.L., Xu W.D., Ji X.L., Ni J. (2017). IL-23R Mutation Is Associated with Ulcerative Colitis: A Systemic Review and Meta-Analysis. Oncotarget.

