# Supplementary material for: Paradoxical Psoriasis Induced by Anti-TNFα Treatment: Evaluation of Disease-Specific Clinical and Genetic Markers
Source: Int J Mol Sci. 2020 Oct 23;21(21):7873. doi: 10.3390/ijms21217873 (PMC7660646; doi:10.3390/ijms21217873)
Supplement: Supplementary file 1 [file ijms-21-07873-s001.pdf]

SUPPLEMENTARY TABLES

Table S1: Clinical-pathologic characteristics of IBD and psoriatic patients with PP treated with Adalimumab.

**Characteristics**[a]	**IBD patients with PP (n=11)**	**Psoriatic patients with PP (n=23)**	*p value**
**Sex**			
Male	3 (27.3%)	9 (39.1%)	
Female	8 (72.7%)	14 (60.9%)	0.06
**Number of psoriatic lesion locations**			
1	1 (9.1%)	14 (73.7%)	
2	3 (27.3%)	2 (10.5%)	
3 or more	7 (63.6%)	3 (15.8%)	**0.003**
**Psoriatic lesions locations**			
Lower limbs	3 (9.7%)	5 (18.5%)	
Upper limbs	1 (3.2%)	3 (11.1%)	
Armpits	4 (12.9%)	0 (0.0%)	
Nipple	1 (3.2%)	0 (0.0%)	
Scalp	8 (25.8%)	1 (3.7%)	
Genitals	4 (12.9%)	2 (7.4%)	
Hands	0 (0.0%)	4 (14.8%)	
Umbilicus	3 (9.7%)	0 (0.0%)	
Retroauricular	4 (12.9%)	3 (11.1%)	
Back	1 (3.2%)	2 (7.4%)	
Trunk	1 (3.2%)	2 (7.4%)	
Nails	0 (0.0%)	1 (3.7%)	
Face	1 (3.2%)	2 (7.4%)	
Suberitrodermic (diffuse cutaneous involvement)	0 (0.0%)	2 (7.4%)	**0.04**
**Pruritus**			
No	6 (50.0%)	4 (18.2%)	
Yes	6 (50.0%)	18 (81.8%)	*0.05*
**Number of months from the beginning of therapy to the development of the paradoxical effect** (Mean ± standard error)	9 (± 1.9)	40.8 (±8.3)	**0.009**

*In bold statistically significant results

[a]Some data for each pathologic characteristic are not available

Abbreviations: IBD: Inflammatory bowel diseases; PP: Paradoxical psoriasis.

Table S2: Distribution of psoriatic patients with and without PP according to genotype frequencies of the six SNPs analyzed.

Gene	SNP	Genotype	Psoriatic patients with PP (n=23)	Psoriatic patients without PP (n=70)	p value*
			n (%)	n (%)	
HLA-Cw06	**rs10484554**	C/C	9 (39.1%)	27 (38.6%)	0.7
		C/T	9 (39.1%)	33 (47.1%)	
		T/T	5 (21.7%)	10 (14.3%)	
		Per-allele	OR 1.1 (95%CI 0.6-2.2) p=0.7		
IL23R	**rs11209026**	G/G	22 (95.6%)	60 (85.7%)	0.2
		G/A	1 (4.4%)	10 (14.3%)	
		A/A	0 (0.0%)	0 (0.0%)	
		Per-allele	OR 0.3 (95%CI 0.03-2.3) p=0.2		
IL23R	**rs10789229**	T/T	11 (47.8%)	29 (41.4%)	0.7
		T/C	8 (34.8%)	31 (44.3%)	
		C/C	4 (17.4%)	10 (14.3%)	
		Per-allele	OR 0.9 (95%CI 0.5-1.8) P=0.8		
TNFa	**rs1799964**	T/T	13 (56.5%)	42 (60.0%)	0.2
		T/C	7 (30.4%)	26 (37.1%)	
		C/C	3 (13.0%)	2 (2.9%)	
		Per-allele	OR 1.4 (95% CI 0.7-3.1) p=0.3		
TNFa	**rs1800629**	G/G	21 (91.3%)	64 (91.4%)	0.6
		G/A	1 (4.4%)	5 (7.1%)	
		A/A	1 (4.3%)	1 (1.4%)	
		Per- allele	OR 1.2 (95% CI 0.4-4) p=0.7		
IFIH1	**rs1990760**	T/T	8 (34.8%)	24 (34.3%)	0.7
		T/C	11 (47.8%)	28 (40.0%)	
		C/C	4 (17.4%)	18 (25.7%)	
		Per-allele	OR 0.8 (95% CI 0.5-1.6) p=0.6		

*In bold statistically significant results

Abbreviations: PP: Paradoxical psoriasis; IFIH1: Interferon induced with helicase C domain 1; TNF-α: Tumor necrosis factor-alpha.

Table S3: Distribution of IBD patients with PP and psoriatic patients without PP treated with Adalimumab according to genotype frequencies of the six SNPs analyzed.

**Gene**	**SNP**	**Genotype**	**IBD patients with PP (n=11)**	**Psoriatic patients without PP (n=70)**	**p value***
			**N (%)**	**N (%)**	
HLA-Cw06	**rs10484554**	C/C	9 (81.8%)	27 (38.6%)	**0.02**
		C/T	2 (18.2%)	33 (47.1%)	
		T/T	0 (0.0%)	10 (14.3%)	
		Per-allele	OR 0.2 (95% CI 0.04-0.7) **p=0.02**		
IL23R	**rs11209026**	G/G	9 (81.8%)	60 (85.7%)	0.73
		G/A	2 (18.2%)	10 (14.3%)	
		A/A	0 (0.0%)	0 (0.0%)	
		Per-allele	OR 1.33 (95% CI 0.3-7.1) p=0.7		
IL23R	**rs10789229**	T/T	8 (72.7%)	29 (41.4%)	**0.02**
		T/C	0 (0.0%)	31 (44.3%)	
		C/C	3 (27.3%)	10 (14.3%)	
		Per-allele	OR 0.7 (95% CI 0.3-1.8) p=0.4		
TNFa	**rs1799964**	T/T	4 (36.4%)	42 (60.0%)	0.06
		T/C	5 (45.5%)	26 (37.1%)	
		C/C	2 (18.1%)	2 (2.9%)	
		Per-allele	OR 2.8 (95% CI 1-7.7) **p=0.05**		
TNFa	**rs1800629**	G/G	11 (100%)	64 (91.4%)	0.6
		G/A	0 (0.0%)	5 (7.1%)	
		A/A	0 (0.0%)	1 (1.4%)	
		Per-allele	-		
IFIH1	**rs1990760**	T/T	4 (36.3%)	24 (34.3%)	0.8
		T/C	5 (45.5%)	28 (40.0%)	
		C/C	2 (18.2%)	18 (25.7%)	
		Per-allele	OR 0.8 (95% CI 0.4-2) p= 0.7		

*In bold statistically significant results

Abbreviations: IBD: Inflammatory bowel diseases; PP: Paradoxical psoriasis; IFIH1: Interferon induced with helicase C domain 1; TNF-α: Tumor necrosis factor-alpha.
